# Genomic differences between type strain PG1 and field strains of *Mycoplasma mycoides* subsp. *mycoides* small-colony type

**DOI:** 10.1016/j.ygeno.2006.06.018

**Published:** 2006-11

**Authors:** Daniela F. Bischof, Edy M. Vilei, Joachim Frey

**Affiliations:** Institute of Veterinary Bacteriology, University of Bern, Länggass-Strasse 122, Postfach, CH-3001 Bern, Switzerland

**Keywords:** Genomic plasticity, *M. mycoides* subsp. *mycoides* SC, Contagious bovine pleuropneumonia, Repeated segments

## Abstract

The recently accomplished complete genomic sequence analysis of the type strain PG1 of *Mycoplasma mycoides* subsp. *mycoides* small-colony type revealed four large repeated segments of 24, 13, 12, and 8 kb that are flanked by insertion sequence (IS) elements. Genetic analysis of type strain PG1 and African, European, and Australian field and vaccine strains revealed that the 24-kb genetic locus is repeated only in PG1 and not in other *M. mycoides* subsp. *mycoides* SC strains. In contrast, the 13-kb genetic locus was found duplicated in some strains originating from Africa and Australia but not in strains that were isolated from the European outbreaks. The 12- and 8-kb genetic loci were found in two and three copies, respectively, in all 28 strains analyzed. The flanking IS elements are assumed to lead to these tandem duplications, thus contributing to genomic plasticity. This aspect must be considered when designing novel diagnostic approaches and recombinant vaccines.

*Mycoplasma mycoides* subsp. *mycoides* small-colony type (SC) is the etiological agent of contagious bovine pleuropneumonia (CBPP), a severe, highly contagious respiratory disease of cattle and buffalo. The disease is endemic on the African continent while, in other parts of the world where severe epidemics occurred in the past, a drastic decimation of the cattle population was realized and as consequence the disease was successfully eradicated [1]. Based on protein analysis [2], *M. mycoides* subsp. *mycoides* SC strains were reported to be homogeneous. However, genetic variations are known to occur within *M. mycoides* subsp. *mycoides* SC as evidenced by restriction fragment length polymorphism [3], [4], by IS*1296* and IS*1634* fingerprinting [5], [6], [7], and by multilocus sequence analysis [8]. IS*1296* fingerprinting allowed differentiation of the cluster of *M. mycoides* subsp. *mycoides* SC strains of the recent outbreaks at the end of the last century in Europe from strains originating from the African and Australian continents [5]. Furthermore, all *M. mycoides* subsp. *mycoides* SC strains isolated in Europe since 1990 revealed a major chromosomal deletion of 8.84 kb, including genes of the glycerol ABC transporter operon and the lipoprotein gene *lppB* [9]. These studies demonstrated that the genome of *M. mycoides* subsp. *mycoides* SC has a higher degree of plasticity than expected. The relatively strong genomic variability must also be taken into consideration when assessing the stability and safety of live vaccine strains and monospecific antigenic diagnostic tests. In addition to genomic variations, a variable surface antigen, Vmm, which undergoes reversible phase variation, has been discovered in *M. mycoides* subsp. *mycoides* SC [10]. This mechanism might be involved in the specific host–tissue interaction at various stages of infection or may play a role in escaping the host's immune defense as shown for other *Mycoplasma* species [11], [12].

The complete genome sequence of *M. mycoides* subsp. *mycoides* SC type strain PG1 has recently been determined [13] and is expected to significantly contribute to the research on molecular mechanisms of pathogenicity of this *Mycoplasma* species. The genome of *M. mycoides* subsp. *mycoides* SC has a high degree of repetitive sequences compared to those of other bacteria. In total, the repetitive sequences in *M. mycoides* subsp. *mycoides* SC constitute 29% of the genome. *M. mycoides* subsp. *mycoides* SC is known to have the highest density of insertion sequences (IS) among bacterial genomes. Three IS elements are known in *M. mycoides* subsp. *mycoides* SC: IS*1296* [6], which has a size of 1485 bp and is present in 28 copies, IS*1634* [7], which has a length of 1872 bp and is present in 60 copies, and IS*Mmy1* [14], which is 1670 bp in length and is present in 9 copies. The IS elements are evenly distributed across the genome except for three large IS-free regions, which are located at nucleotide (n.t.) positions 285,937 to 363,559, 471,574 to 592,871 and 828,541 to 881,279 on the genome sequence (GenBank/EMBL Accession No. NC_005364) [13].

In addition to the IS elements, the genomic sequence also revealed large repeated genomic segments which themselves are flanked by IS elements [13]. These large repeats contain important genes such as *lppQ*, encoding lipoprotein Q that induces the main antigenic response in naturally and experimentally infected cattle [15], *nox*, which encodes the NADH oxidase [13], *ptsG*, which encodes the permease of the phosphoenolpyruvate:glucose phosphotransferase system (glucose PTS) [16], *phnB, phnC,* and *phnD,* which encode alkylphosphonate ABC transporter components [13], and *oppF*, which encodes an oligopeptide ABC transporter [13] (Fig. 1). This finding raises the question whether these genes are repeated due to their high importance in the life cycle or in the molecular mechanism of pathogenicity of *M. mycoides* subsp. *mycoides* SC. It has to be noticed that bacterial type strains often have a genetic arrangement that does not correlate to that of field strains. Moreover, they frequently are less pathogenic than field strains of the same species. This has, e.g., recently been observed with *Aeromonas salmonicida* subsp. *salmonicida*, where the type strain lacks a large 140-kb virulence plasmid and hence is avirulent [17]. Also the type strain PG1 of *M. mycoides* subsp. *mycoides* SC is assumed to be significantly less pathogenic than field strains [18]. We therefore investigated African and European field strains and vaccine strains of *M. mycoides* subsp. *mycoides* SC for the presence of the four major large duplicated segments that are found in type strain PG1.

**Fig. 1:**
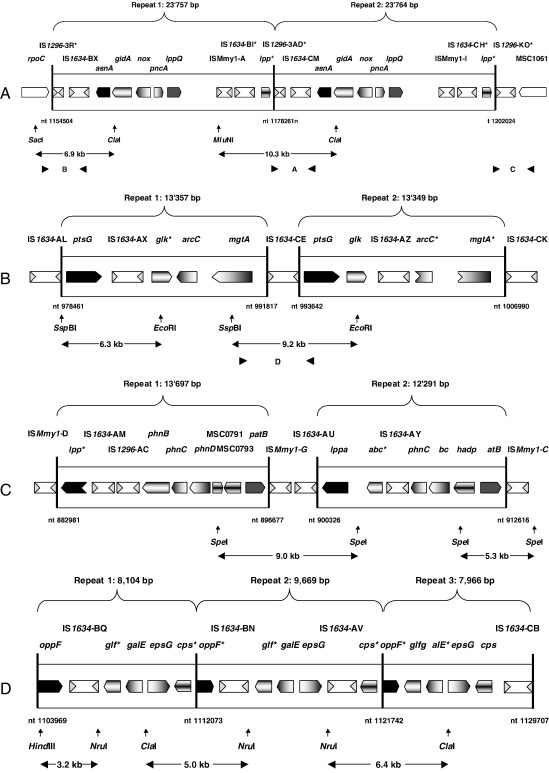
Genetic map of the 24-kb repeat locus (A), the 13-kb repeat locus (B), the 12-kb repeat locus (C) and the 8-kb repeat locus (D) in type strain PG1 of *M. mycoides* subsp. *mycoides* SC. Horizontal black arrowheads indicate position of the various oligonucleotide primers and the gaps between two paired arrowheads show the amplified PCR fragments A–D. The fragments A–D relate to the PCR products shown in Fig. 2, Fig. 3. Restriction sites for *Sac*I, *Cla*I, *Mlu*NI, *Ssp*BI, *Eco*RI, *Spe*I, *Hin*dIII, and *Nru*I are indicated by vertical arrows. Double-headed arrows with integrated fragment lengths indicate the generated digestion fragments that react positively with DNA probes on Southern blots. The solid vertical bars show the borders of the repeated segments and the corresponding figures give the exact position in nucleotides (nt) referring to the genomic sequence NC_005364. The exact number of basepairs of the individual repeated segments is indicated on each brace. Genes are represented by pentagons (arrowed boxes). Negatively arrowed boxes or chevrons indicate truncated genes. In each panel, the same shading was used for the same genes. IS elements are indicated by white boxes and their inverted repeats by gray triangles. Gene abbreviations: *rpoC*, DNA-directed RNA polymerase beta' chain; *asnA*, aspartate-ammonia ligase; *gidA*, glucose inhibited division protein A; *nox*, NADH oxidase; *pncA*, pyrazinamidase/nicotinamidase; *lppQ*, prolipoprotein Q; *lpp*, putative variable lipoprotein; *ptsG*, PTS system, glucose-specific IIBC component; *glk*, glucokinase; *arc*, carbamate kinase; *mgtA*, Mg^2+^-transport ATPase; *phnB*, alkylphosphonate ABC transporter, permease component; *phnC*, alkylphosphonate ABC transporter, ATP-binding component; *phnD*, alkylphosphonate ABC transporter, substrate binding component; *patB*, aminotransferase (PLP-dependent); *abc*, ABC transporter, permease component; *had*, hydrolase of the HAD family; *oppF*, oligopeptide ABC-transporter; *glf*, UDP-galactopyranose mutase; *galE*, UDP-glucose 4-epimerase; *epsG*, glycosyltransferase; *cps*, glycosyltransferase. Asterisks (*) indicate truncated or nonfunctional genes or genetic elements.

## Results

### In silico analysis of the genome of *M. mycoides* subsp. *mycoides* SC

Analysis of the genomic sequence of *M. mycoides* subsp. *mycoides* SC was carried out by using the software MolliGen 1.5 (http://www.cbi.labri.fr/outils/molligen/). Four regions containing long repeats of 24, 13, 12, and 8 kb were found. They are located in the vicinity of the origin of replication *oriC*, all on the same side with respect to *oriC*.

The 24-kb repeats are located at n.t. positions 1,154,504 to 1,178,260 and 1,178,261 to 1,202,024 based on the coordinates of the genomic sequence (GenBank/EMBL Accession No. NC_005364) and are flanked by IS elements IS*1296* (Fig. 1A). The genomic sequence data reveal the two 24-kb repeated segments to be identical with the exception of 7 additional bp in a noncoding segment of the second repeat. Another few minor differences between the two repeats as reported in the genomic DNA sequence NC_005364 could not be confirmed by resequencing these loci and this might be due to initial sequencing errors. The 13-kb repeats are located at n.t. positions 978,461 to 991,817 and 993,642 to 1,006,990 and are flanked by IS elements IS*1634* (Fig. 1B). They have sizes of 13,357 and 13,349 bp, respectively, and are only partially identical due to the fact that two genes, *arcC* and *mgtA*, are truncated in the second copy of the 13-kb segment and one gene, *glk,* seems to be nonfunctional in the first copy (Fig. 1B). The 12-kb repeats (Fig. 1C) are located at n.t. positions 882,981 to 896,677 and 900,326 to 912,616 and are flanked by IS elements IS*Mmy1*. The size of the first segment is 13,697 bp, whereas the size of the second segment is 12,291 bp. This difference of approximately 1400 bp is due to the presence of IS element IS*1296-*AC, which is present only in the first segment and not in the second segment. The three 8-kb repeats (Fig. 1D) are located at n.t. positions 1,103,969 to 1,112,072, 1,112,073 to 1,121,741 and 1,121,742 to 1,129,707 and harbor the IS elements IS*1634*. They have sizes of 8104, 9669, and 7966 bp, respectively. The three 8-kb segments show major differences: the first segment contains the genes *oppF*, glf*, galE, *epsG,* and cps* with the genes glf* and cps* being truncated. The second segment contains the genes oppF*, glf*, galE, epsG, and cps*, where the genes oppF*, glf* and cps* are truncated. The third segment contains the genes oppF*, glf, galE*, *epsG,* and *cps.* In this segment the genes oppF* and galE* are truncated (Fig. 1D).

### Genetic differences in type strain, vaccine, and field strains

**Fig. 2:**
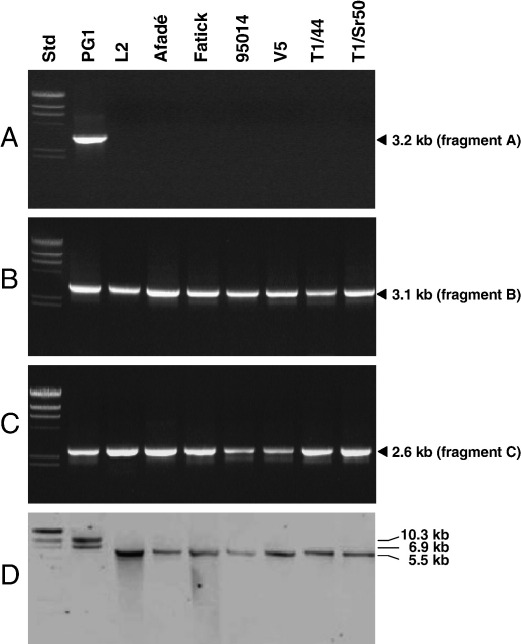
PCR and Southern blot results of a selection of eight strains of *M. mycoides* subsp. *mycoides* SC for investigation of the 24-kb repeats. PCR was performed with 50 ng of genomic DNA using oligonucleotide primers: (A) MmmSC_23760fwd and MmmSC_1rev to obtain fragment A of Fig. 1A; (B) MmmSC_rpoC_fwd and MmmSC_MSC1011rev to obtain fragment B of Fig. 1A; and (C) MmmSC_23760fwd and MmmSC_MSC1061rev to obtain fragment C of Fig. 1A. (D) Southern blot hybridization of *Cla*I-, *Sac*I-, and *Mlu*NI-digested DNA hybridized to the *asnA* probe. Std, molecular mass standards: 23.1, 9.4, 6.6, 4.4, 2.3, and 2.0 kb.

We investigated the presence of the long tandem repeats of the 24-kb segment in PG1, in 1 old European, 11 recent European, 8 African, and 3 Australian field strains and in 3 vaccine strains (Table 1). Fig. 1A reveals that primers MmmSC_23760fwd and MmmSC_1rev (Table 2) should amplify a 3.2-kb fragment (Fig. 1A, PCR fragment A) in the case of the presence of a tandem repeat. As shown in Fig. 2A for a selection of 8 strains, only type strain PG1 gave an amplification product while the field and vaccine strains tested presented no amplicons. As a positive control for this genomic segment, PCRs using the primer pair MmmSC_rpoC_fwd/MmmSC_MSC1011rev (Table 2) were made and resulted in the amplification of a 3.1-kb fragment (Fig. 1A, PCR fragment B) in all strains tested and as shown exemplarily for the 8 strains in Fig. 2B. The intergenic region between *rpoC* and IS*1296*-3R from fragment B of these selected strains was analyzed by DNA sequencing with the primers MmmSC_rpoC_fwd and MmmSC_IS1296rev1 (Table 2) and showed the same nucleotide sequence as that found in type strain PG1. As a further positive control for the other end of this genomic segment, PCRs using the primer pair MmmSC_23760fwd/MmmSC_MSC1061rev (Table 2) were performed and resulted in the amplification of the expected 2.6-kb fragment (Fig. 1A, PCR fragment C) in all strains tested. This is shown for the 8 selected strains in Fig. 2C. Sequence analysis with the primers MmmSC_23760fwd and MmmSC_IS1296rev2 (Table 2) of the intergenic region between *lpp* and IS*1296*-KO from fragment C of these strains revealed the same nucleotide sequence as that from type strain PG1. To verify the PCR results, Southern blot hybridization of *Cla*I-, *Mlu*NI-, and *Sac*I-digested genomic DNA was performed with a gene probe for *asnA* (Fig. 1A) that was produced by PCR DIG-labeling using the primer pair MmmSC_asnA3′/MmmSC_asnA5′ (Table 2). As shown in Fig. 2D, the Southern blot resulted in the two expected bands of 6.9 kb (*Sac*I–*Cla*I) and 10.3 kb (*Mlu*NI–*Cla*I) for type strain PG1. Field strains of *M. mycoides* subsp. *mycoides* SC and the vaccine strains T1/44, T1/Sr50, and V5 revealed only one copy of this segment and it has to be noted that the fragment hybridizing to *asnA* in these strains is approximately 1.4 kb smaller than the *Sac*I–*Cla*I fragment of PG1 (Fig. 2D). This difference could be due to the lack of an IS element in this segment and was not analyzed further in detail.

**Table 1:** *Mycoplasma* strains used in this study and number of large genomic repeats detected

Strain	Origin	Year isolated	Host	Repeats			
				24-kb	13-kb	12-kb	8-kb
PG1^a^	Unknown	1931	Cattle/type strain	2	2	2	3
2059^b^	Spain	1984	Cattle/lung	1	1	2	3
B773/125^c^	Portugal	1991	Cattle/semen	1	1	2	3
B820/123^c^	Portugal	1991	Cattle/preputial wash	1	1	2	3
C305^c^	Portugal	1993	Goat/lung	1	1	2	3
C425^c^	Portugal	1993	Goat/lung	1	1	2	3
PO 2^d^	France	1980	Cattle/lung	1	1	2	3
2022^c^	France	1984	Cattle/lung	1	1	2	3
L2e,^f^	Italy	1993	Cattle/lung	1	1	2	3
402^c^	Italy	1990	Cattle/lung	1	1	2	3
6479^c^	Italy	1992	Cattle/lung	1	1	2	3
PO 1967^d^	France	1967	Cattle/lung	1	1	2	3
Afadé^d,f^	Cameroon^g^	1968	Cattle	1	2	2	3
Fatick^d^	Senegal	1968	Cattle	1	2	2	3
B17^d^	Chad	1967	Zebu	1	1	2	3
7721^d^	Mauritania	1977	Cattle	1	1	2	3
9050-529/1^d^	Ivory Coast	1990	Cattle	1	2	2	3
Asmara^d^	Eritrea	1970	Cattle	1	1	2	3
91130^d^	Central African Republic	1991	Cattle	1	1	2	3
C11^d^	Chad	1962	Cattle	1	1	2	3
94111^d^	Rwanda	1994	Cattle	1	2	2	3
95014^d^	Tanzania	1995	Cattle	1	2	2	3
T1/44^d^	Tanzania	1952	Cattle/vaccine strain	1	2	2	3
T1/Sr50^d^	Tanzania	1952	Cattle/vaccine strain	1	2	2	3
Gladysdale^h^	Australia		Cattle	1	2	2	3
R575^h^	Australia	1965–1968	Cattle	1	1	2	3
DVZ^h^	Australia	1965	Cattle	1	2	2	3
V5^h^	Australia	1965–1968	Cattle/vaccine strain	1	1	2	3

a National collection of type cultures (NCTC), PHLS, London, UK.

b Laboratoire de Pathologie Bovine, Lyon, France.

c Laboratorio Nacional de Veterinaria, Lisbon, Portugal.

d CIRAD-EMVT, Montpellier, France.

e Institute for Veterinary Bacteriology, University of Bern, Bern, Switzerland.

f Reference strains representing the European-cluster, respectively, African-cluster strains.

g Isolated at Farcha Laboratory, N'Djaména, Chad from a bovine from Afadé, Northern Cameroon.

h Australian Animal Health Laboratory, Geelong, Victoria, Australia.

**Table 2:** Oligonucleotide primers

Primer	Sequence (5′–3′)	Nucleotide position^a^ (range)
*24-kb repeats*		
MmmSC_rpoC_fwd	GCGCTGTTGCTATAATTTCTG	1153795–1153815
MmmSC_IS1296rev1	CAGGGATTTCATTATCAGCAC	1154820–1154800; 1178577–1178557; 1202341–1202321^b^
MmmSC_MSC1011rev	CCTACGTTTCCCAGTTTAAGC	1156846–1156826; 1180603–1180583^b^
MmmSC_1rev	TGATGATGTTTGACAAGAATATG	1157471–1157449; 1181228–1181206
MmmSC_asnA5′	CTTGTACTTCACCAATATGAGC	1157867–1157888; 1181624–1181645
MmmSC_asnA3′	CAAACTCAACAAGCTATTCAAG	1158758–1158737; 1182515–1182494
MmmSC_23760fwd	AATAATAATGCAGTTCCACAATC	1178055–1178077; 1201819–1201841
MmmSC_IS1296rev2	TGATGCACTGATGTAACAGAG	1202533–1202513^b^
MmmSC_MSC1061rev	AGAACAGGACCCGTACTATC	1204456–1204437

*13-kb repeats*		
MmmSC_ptsG5′	GGCATTTGCTGCTAATATTATG	978849–978870; 994029–994050
MmmSC_ptsG_rev	TGCTAAAGCTACAGAAATACAG	979077–979056; 994257–994236
MmmSC_ptsG3′	CAACTTTCATTTCACTTACAGG	980827–980806; 996007–995986
MmmSC_MSC0861	TGGATTAAGTTTAGATGTATTAAG	981581–981604; 996761–996784
MmmSC_glkR	CAAGTGCAGCAGCATTAA	984475–984458; 997777–997760
MmmSC_arcC3′	TTTAACAAATTTAGTTCCACTTAG	985163–985186; 998465–998488
MmmSC_arcC5′	GTAATTGCAATTGGTGGAAATG	986080–986059; 1001263–1001242
MmmSC_mgtA2F	CAAAATAATAACCCGCAATTGC	987867–987888; 1003049–1003070
MmmSC_mgtA2R	GGATTTGCATCATTTTTAGATAC	988626–988604; 1003807–1003785
MmmSC_mgtA_fwd	GAGTTAATCCAACTGCAACAG	989253–989273; 1004434–1004454

*12-kb repeats*		
MmmSC_patB_F	TGAATAAGTTTAAATCTGAGTTTG	894982–895005; 910921–910944
MmmSC_patB_R	AGATTTTTAGTTTTTCAACTAATTG	896154–896130; 912093–912069

*8-kb repeats*		
MmmSC_oppFfwd	GGTTAATAAAGTACAAATGATTTTTC	1104343–1104368; 1112075–1112100; 1121744–1121769
MmmSC_oppFrev	TTCAAATTTATACACTTCTAGTTC	1105276–1105253; 1113008–1112985; 1122674–1122651^b^

*TaqMan*		
lppQTM-L	AATAATCAACAAAAAAAAGAGCAAGTAAGTAA	1166019–1166050; 1189776–1189807
lppQTM_FT	ACATCTTGTTTTTGTCACTCATTTTTTGGTTCAATTTT	1166113–1166076; 1189870–1189833
lppQTM-R	TAGCCCTTTTATTTTTAGTAATGCTTGTAA	1166144–1166115; 1189901–1189872
glpOTM-L	CTGAAGCAGGTATTGTAAATTCTGTAGTATT	294492–294462
glpOTM-FT	CATTCATGGAAAAGGAGTTATTGTTGCTCCAA	294449–294418
glpOTM-R	AAAGCAGTTGGTCCTACCATAACTCT	294381–294406

a Based on the nucleotide sequence NC_005364.

b Primers anneal also to homologous sequences out of the repeats investigated in this study. Only positions in the relative repeats are listed.

TaqMan analysis was performed to confirm the existence of the 24-kb tandem repeats in *M. mycoides* subsp. *mycoides* SC type strain PG1 and the presence of a unique 24-kb segment in all other strains. The genes *lppQ* (located in the 24-kb segment) and *glpO* (selected as an endogenous control; a single copy is present in all strains) were chosen as targets for the real-time PCR. The standard curves (Ct values vs log copy number) generated by 10-fold dilutions of DNA from two different *M. mycoides* subsp. *mycoides* SC strains were linear (*r*^2^ values of > 0.999) over a range of 7–8 log units for both target genes, with slopes very close to − 3.32 and efficiencies of ∼ 1 (not shown). TaqMan analysis of various *M. mycoides* subsp. *mycoides* SC strains confirmed the existence of two copies of *lppQ* in type strain PG1 only, as the value 2^(1+ΔΔCt)^ was always ∼ 1 for all other strains analyzed (Table 3), indicating that they contained only one copy of the *lppQ* gene.

**Table 3:** TaqMan analysis for determination of copies of the *lppQ* gene

Strain	Ct_lppQ_^a^	Ct_glpO_^a^	ΔCt^b^	ΔΔCt^c^	2^(1+ΔΔCt)^^d^	*lppQ* copies
PG1	16.69	18.50	1.81	0.00	2.00	2
C305	17.00	17.86	0.86	− 0.95	1.04	1
PO 2	16.33	17.16	0.83	− 0.98	1.01	1
2022	16.51	17.25	0.74	− 1.07	0.95	1
L2	16.46	17.23	0.77	− 1.04	0.97	1
Afadé	16.98	17.90	0.92	− 0.89	1.08	1
Fatick	16.28	16.99	0.71	− 1.10	0.93	1
B17	16.33	17.24	0.91	− 0.90	1.07	1
95014	16.66	17.43	0.77	− 1.04	0.97	1
T1/44	16.06	16.88	0.82	− 0.99	1.01	1
T1/Sr50	16.84	17.71	0.87	− 0.94	1.04	1
Gladysdale	16.88	17.76	0.88	− 0.93	1.05	1
R575	16.16	16.89	0.73	− 1.08	0.95	1
DVZ	16.19	16.93	0.74	− 1.07	0.95	1
V5	16.41	17.14	0.73	− 1.08	0.95	1

a The values are the means of two or three independent measurements.

b Ct_glpO_–Ct_lppQ_ (normalization of endogenous control).

c ΔCt_strain_–ΔCt_PG1_ (normalization to PG1).

d Ratio between copies of *lppQ* and copies of *glpO*.

**Fig. 3:**
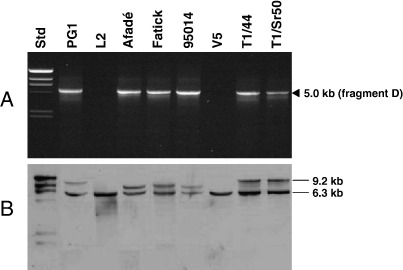
PCR and Southern blot results of a selection of eight strains of *M. mycoides* subsp. *mycoides* SC for investigation of the 13-kb repeats. (A) PCR performed with 50 ng of genomic DNA using oligonucleotide primers MmmSC_mgtA_fwd and MmmSC_ptsG_rev to obtain fragment D of Fig. 1B. (B) Southern blot hybridization of *Eco*RI- and *Ssp*BI-digested DNA hybridized to the *ptsG* probe. Std, molecular mass standards.

The presence of the 13-kb repeat was investigated by PCR amplification using oligonucleotide primers MmmSC_mgtA_fwd and MmmSC_ptsG_rev (Table 2). A 5.0-kb fragment (Fig. 1B, PCR fragment D) was expected from strains carrying the 13-kb segment as a tandem repeat and no amplification was expected for strains carrying a single copy of this segment. Surprisingly, some strains gave an amplification product and some did not, indicating that in some strains the 13-kb region was duplicated as a tandem repeat but not in others (Fig. 3A, Table 1). Southern blot hybridization of *Eco*RI- and *Ssp*BI-digested genomic DNA with a gene probe for *ptsG,* which was made by using the primer pair MmmSC_ptsG3′/MmmSC_ptsG5′ (Table 2), revealed the two expected fragments of 9.2 and 6.3 kb in type strain PG1 (Fig. 3B). The patterns on Southern blots from the field and vaccine strains confirmed the presence or absence of two copies of the 13-kb segment as revealed by PCR analysis. It has, however, to be noted that the upper of the two hybridizing fragments of the field strains, but not of the vaccine strains T1/44 and T1/Sr50, was somewhat smaller than that from PG1, which is assumed to be due to the formation of a minor deletion or loss of an IS element in this segment. The three genes *glk*, *arcC,* and *mgtA*, which are functional in only one of the 13-kb repeats of PG1 and are mutated in the other repeat (Fig. 1B), were amplified with the primer pair MmmSC_MSC0861/MmmSC_mgtA2R (Table 2) from the European strain L2, which has only one copy of the 13-kb segment (Table 1), and the DNA stretches that differ in PG1 were sequenced in L2 with primers MmmSC_MSC0861, MmmSC_glkR, MmmSC_arcC3′, MmmSC_arcC5′, MmmSC_mgtA2F, and MmmSC_mgtA2R (Table 2). Sequence analysis showed that the gene *glk* of L2 corresponded to the functional copy of *glk* in the second repeat of the 13-kb segment in PG1. The sequences of the genes *arcC* and *mgtA* of L2 correspond to the complete functional open reading frames of the first copy of the 13-kb segment in PG1 (Fig. 1B).

**Fig. 4:**
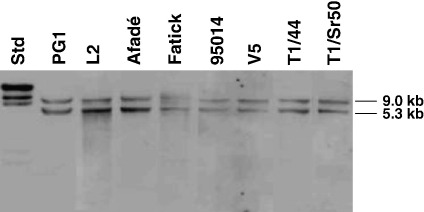
Southern blot hybridization of *Spe*I-digested DNA of a selection of eight *M. mycoides* subsp. *mycoides* SC strains hybridized to the *patB* probe for investigation of the 12-kb repeats. Std, molecular mass standards.

The duplication of the 12-kb repeat was investigated by Southern blot hybridization only, since the nature of this tandem repeat did not allow an accurate PCR strategy to verify the duplication (Fig. 1C). Southern blot hybridization of *Spe*I-digested genomic DNA with a gene probe for *patB*, which was made using the primer pair MmmSC_patB_F/MmmSC_patB_R (Table 2), revealed the presence of the two expected fragments of 9.0 and 5.3 kb in PG1 and showed their presence in all other strains investigated (Fig. 4; Table 1).

**Fig. 5:**
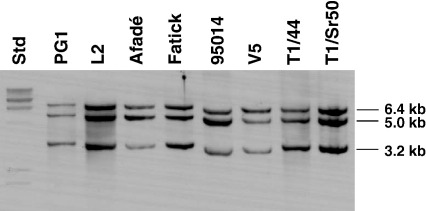
Southern blot hybridization of *Hin*dIII-, *Cla*I-, and *Nru*I-digested DNA of a selection of eight *M. mycoides* subsp. *mycoides* SC strains hybridized to the *oppF* probe for investigation of the 8-kb repeats. Std, molecular mass standards.

The presence of the triple-repeated segment of 8 kb was analyzed by Southern blot hybridization of *Hin*dIII-, *Cla*I-, and *Nru*I-digested genomic DNA with a gene probe for *oppF* (Fig. 1D), which was made by PCR amplification using the primer pair MmmSC_oppFfwd/MmmSC_oppFrev (Table 2). Three bands of 6.4, 5.0, and 3.2 kb were expected for PG1. As shown in Fig. 5 and Table 1, all strains showed the expected hybridization pattern containing these three bands demonstrating the existence of this tripled repeat in all strains analyzed.

### Stability of large tandem repeats in *M. mycoides* subsp. *mycoides* SC

**Fig. 6:**
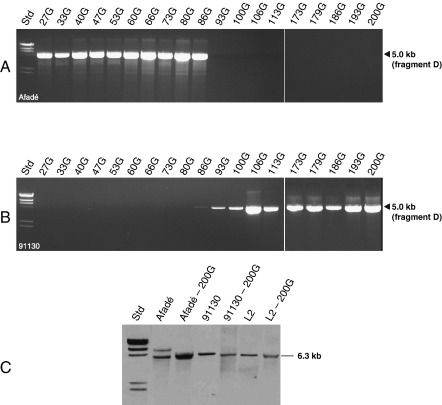
Plasticity of the 13-kb repeats. PCR performed using oligonucleotide primers MmmSC_mgtA_fwd and MmmSC_ptsG_rev to obtain fragment D of Fig. 1B with lysates of passaged (up to 200 generations) *M. mycoides* subsp. *mycoides* SC strains: (A) Afadé and (B) 91130. (C) Southern blot hybridization of *Eco*RI- and *Ssp*BI-digested DNA of strains Afadé, 91130, and L2 after passages 0 or 30 (corresponding to 200 generations, 200G) hybridized to the *ptsG* probe. Std, molecular mass standards.

In vitro stability of *M. mycoides* subsp. *mycoides* SC strains with respect to the tandem repeated segment was tested by growing three selected strains in standard mycoplasma medium. Two strains, L2 and 91130, which possess only one copy of the 13-kb segment, and one strain, Afadé, which possesses two copies of the 13-kb segment, were passaged in growth medium by successive dilution for a total of 200 generations. Samples from cultures with various generations were then lysed and PCR amplification was performed by using oligonucleotide primers MmmSC_mgtA_fwd and MmmSC_ptsG_rev (Table 2) to reveal the presence or absence of the 13-kb tandem repeat. PCR analysis of strain Afadé showed amplicons of 5.0 kb (Fig. 1B, PCR fragment D) only until the 86th generation when they faded and vanished in the following generations (Fig. 6A), indicating the loss of one of the 13-kb repeats. On the other hand, in strain 91130 the PCR amplification product of 5.0 kb appeared after 86 generations and remained until the 200th generation (Fig. 6B). This PCR fragment from strain 91130, which spans the DNA portion harboring part of *mgtA* at the end of repeat 1, IS*1634*-CE, and part of *ptsG* at the beginning of repeat 2, was sequenced (GenBank/EMBL Accession No. AM183254) and found to have some minor discrepancies, i.e., a deletion of 8 n.t. and eight single nucleotide polymorphisms (SNP) compared to the same genomic portion from type strain PG1. The deletion of 8 n.t. and four SNPs were found in intergenic regions, *mgtA* and *ptsG* had one missense SNP each, one silent SNP was found in the transposase gene of IS*1634*-CE, and one SNP was found between *mgtA* and IS*1634*-CE. This allowed the formation of a large open reading frame having almost 100% identity with the hypothetical transmembrane protein MSC_0882 derived from the genome sequence NC_005364 of PG1.

Southern blot hydridization with probe *ptsG* confirmed the loss of one of the copies of the 13-kb tandem repeat in strain Afadé upon passaging (Fig. 6C). However, Southern blot analysis of DNA of the 200th generation of strain 91130 using the *ptsG* probe showed only one copy of the 13-kb segment (Fig. 6C). Thus, although certain DNA areas have been rearranged in strain 91130, as shown by PCR analysis, Southern blot results showed that the segment of 13 kb was not entirely duplicated. PCR analysis and Southern blot analysis revealed no modifications in strain L2 after 200 generations in growth medium (Fig. 6C). Furthermore, no changes in the other genomic segments analyzed in this study could be found in all three strains after 200 generations.

## Discussion

The present analysis of large repeated genomic sections in *M. mycoides* subsp. *mycoides* SC showed that the duplication of the 24-kb segment is peculiar to the type strain PG1 and is not found in field and vaccine strains. We assume that this large duplication in PG1 occurred during the large number of passages in growth medium and was induced by IS*1296*. The 13-kb segment was found duplicated in certain strains from the African and Australian continents but not in strains of the recent European outbreaks, as also observed by Gaurivaud and colleagues [16]. However, our results show that the 13-kb tandem repeat is found only in 9 of 16 representative African/Australian strains and hence cannot be used to differentiate the latter from European strains. Furthermore, we have demonstrated that the 13-kb tandem repeat is rather unstable and can be lost in cultures that are repeatedly passaged in growth medium. Other changes can occur within this 13-kb structure during extended growth in the laboratory, which may produce duplication of portions only of the segment. The 12-kb and the 8-kb repeats seem to be more stable since they were found as duplicates and triplicates, respectively, in all strains investigated.

The duplication of the 24-kb segment seemed to be a rare event, found only in the type strain. These repeats are flanked by copies of IS element IS*1296* that belongs to the IS*3* family [6] and has a low transposition frequency [5]. In contrast, the 13-kb and the 8-kb segments are flanked by or harbor IS*1634* that belongs to the IS*4* family and seems to transpose more frequently than IS*1296* [7]. Hence, IS elements seem to play a central role in genomic rearrangements of *M. mycoides* subsp. *mycoides* SC and seem to determine the likelihood with which such arrangements may occur.

The biological function of the large duplications analyzed in this study is not known. Interestingly, in most cases only one copy of the genes present on the tandem repeats seems to be functional and hence the duplications do not seem to exert gene dosage effects. Therefore, these duplications do not seem to have a direct influence over virulence of *M. mycoides* subsp. *mycoides* SC. However, they must be taken into account when designing molecular diagnostic tools and novel recombinant vaccines. Analyzing the genome of *M. mycoides* subsp. *mycoides* SC, it has to be noticed that the GC skew in *M. mycoides* subsp. *mycoides* SC reveals the putative position of the origin of replication, but it does not follow a normal pattern at the expected *oriC* locus [13]. The fact that the GC skew of *M. mycoides* subsp. *mycoides* SC has an abnormal pattern may be due to rearrangements of the genome that occurred during late evolution of this species. Since the four regions containing the large repeats were all found close to the start of the replication forks, and more precisely on the same side with respect to *oriC*, one might question whether replication in *M. mycoides* subsp. *mycoides* SC is unidirectional, despite the fact that the chromosomal replication in *Mycoplasma* species is reported to be bidirectional [19], [20], [21].

## Materials and methods

### Strains, growth conditions, lysate preparation, and DNA extraction

Strains of *M. mycoides* subsp. *mycoides* SC used in this study and their countries of origin are listed in Table 1. Cultures of *M. mycoides* subsp. *mycoides* SC were made in a standard mycoplasma medium (Axcell Biotechnologies) at 37 °C to a density of 10^8^–10^9^ cells/ml.

Lysed cells were obtained from 1 ml of liquid culture. They were harvested by centrifugation at 8000*g* for 15 min, washed with phosphate-buffered saline (140 mM NaCl, 2.7 mM KCl, 10 mM phosphate buffer, pH 7.4), and resuspended in 150 μl of H_2_O. Then, the cells were lysed by boiling for 10 min and centrifuged at 12,000*g* for 1 min. Aliquots of 100 μl of the supernatants were collected and used for PCR amplification.

For DNA extraction, the cells were harvested by centrifugation at 8000*g* for 10 min, washed three times in TES buffer (10 mM Tris/HCl, 1 mM EDTA, 140 mM NaCl, pH 7.5), and then resuspended in 0.1 volumes of TE buffer (10 mM Tris, 1 mM EDTA, pH 7.5). A sample of 100 μl of resuspended cells was then lysed by addition of 500 μl of GES buffer (5 M guanidium thiocyanate, 100 mM EDTA, 0.5% *N*-lauroylsarcosine) for 10 min at room temperature, cooled on ice, and then mixed with 250 μl of 7.5 M ammonium acetate, pH 7.7. The lysate was then extracted three times with 500 μl of phenol:chloroform:isoamylalcohol (49.5:49.5:1) (Fluka) and the DNA was then precipitated by the addition of 0.7 volumes of isopropanol and centrifugation at 17,500*g* for 15 min at 4 °C in an Eppendorf centrifuge. The pellet was washed three times with 80% ethanol, dried, and resuspended in 100 μl of H_2_O.

### Growth of mycoplasma cultures for high numbers of generations

Cultures of 1 ml of *M. mycoides* subsp. *mycoides* SC were made by inoculation of 50 μl of a frozen stock culture in 1 ml of standard mycoplasma medium and incubation at 37 °C for 3 days. The first passage was accomplished by the addition of 2 ml of medium and further incubation for 3 days. The culture was then passaged in 3 ml of medium at a dilution of 1:100. This procedure was repeated 30 times to achieve as many as 200 generations. After every passage, representing approximately 7 generations as calculated by real-time TaqMan PCR, culture samples were collected for PCR analysis or stored at − 80 °C.

### PCR amplification, Southern blotting, and DNA sequence analysis

Polymerase chain reaction was performed in a DNA thermal cycler Gene Amp 9600 (Applied Biosystems) in a 50-μl reaction mixture [50 mM Tris–HCl, pH 9.2; 1.75 mM MgCl_2_; 16 mM (NH_4_)_2_SO_4_ and 350 μM of each deoxynucleoside triphosphate] containing 300 nM each primer (Table 2), 1.75 U of *Taq* DNA polymerase (Roche Diagnostics), and approximately 50 ng of genomic DNA or 2.5 μl of lysate as template. The mixtures were subjected to 2 min denaturation at 94 °C followed by 35 cycles of amplification with the parameters: 30 s at 94 °C, 30 s at 48 °C, and 1–3 min extension at 72 °C. Digoxigenin-11-dUTP (DIG)-labeled probes were produced by PCR as described above in the presence of 20 μM DIG (Roche Diagnostics).

For the amplification of fragments above 3 kb, PCR was carried out in a 50-μl reaction mixture [5 μl buffer 3 (27.5 mM MgCl_2_ and detergents; Expand Long Template PCR System kit, Roche Diagnostics) and 500 μM each dNTP] containing 300 nM each primer (Table 2), 1.75 U of a mixture of *Taq* and *Pwo* polymerases, and approximately 50 ng of genomic DNA or 2.5 μl of lysate as template. The DNAs were amplified for 35 cycles (30 s of denaturation at 92 °C, 30 s at 48 °C and 3–5 min extension at 68 °C) after one step of 2 min at 92 °C to ensure denaturation.

For Southern blot analysis, genomic mycoplasmal DNA was digested and the fragments were separated by electrophoresis on a 1% (w/v) agarose gel and then transferred onto a positively charged nylon membrane (Roche Diagnostics) following standard protocols [22]. The membrane was probed using DIG-labeled fragments and the probes were detected using phosphatase-labeled anti-DIG antibodies and CDP-*Star* (Roche Diagnostics) as described by the manufacturer.

Amplicons were purified with the High pure PCR product purification kit (Roche Diagnostics) and sequenced with a DNA Sequenator AB 3100 genetic analyzer and the *Taq* DyeDeoxy terminator cycle sequencing kit (Applied Biosystems) using specific oligonucleotide primers (Table 2). Assembling of DNA sequences and alignments of sequenced segments were done using the program Sequencher 4.5 (GeneCodes). Comparisons of DNA sequences and their deduced amino acid sequences with the EMBL/GenBank database were performed using the BLAST programs blastn, blastx, and blastp [23].

### Selection of TaqMan primers and probes

Primer Express 2.0 software (Applied Biosystems) was used to design oligonucleotide primers and fluorogenic probes specific to the genes *lppQ* (located in the 24-kb segment) and *glpO* of *M. mycoides* subsp. *mycoides* SC. The gene *glpO* was selected as an endogenous control as it is present in a single copy in all *M. mycoides* subsp. *mycoides* SC strains [24]. The software found TaqMan primers for both genes (Table 2): lppQTM-L and lppQTM-R for *lppQ* (amplicon of 126 bp) and glpOTM-L and glpOTM-R for *glpO* (amplicon of 112 bp). The corresponding probes lppQTM_FT and glpOTM-FT (Table 2) have the FAM reporter dye and TAMRA quencher affixed on the 5′ and 3′ ends, respectively.

### Quantification of gene copy number by real-time PCR

Reactions for both *lppQ* and *glpO* were performed by using 2.5 μl of DNA, a 900 nM concentration of each TaqMan primer, 300 nM TaqMan probe, and TaqMan Universal PCR Master Mix (Applied Biosystems) in a 25-μl volume. PCRs were run separately on an ABI 7500 instrument (Applied Biosystems) using the following cycling parameters: after one step at 50 °C for 2 min and at 95 °C for 10 min, 50 cycles of denaturation at 95 °C for 15 s, and extension at 60 °C for 1 min were performed. Real-time fluorescence measurements were taken and the cycle number at which the fluorescent signal crossed the default threshold limit (set at 0.062 for *lppQ* and 0.122 for *glpO*) for each sample was recorded (Ct value). Each assay was repeated at least twice.

Standard curves were produced by analyzing 10-fold dilutions of genomic DNAs of *M. mycoides* subsp. *mycoides* SC strains 2022 and L2 (Table 1) containing 0.62 fg to 615.0 ng of 2022 DNA or 0.16 fg to 156.5 ng of L2 DNA, which corresponded to 0.47 to 4.68 × 10^8^ or 0.12 to 1.19 × 10^8^ genome equivalents (geq), respectively, bearing in mind that *M. mycoides* subsp. *mycoides* SC has a genome size of approximately 1.2 Mb [13]. The efficiencies of both TaqMan reactions were calculated considering the slopes of the standard curves and using the formula [10^(− 1/slope)^]−1.

To calculate the number of copies of the *lppQ* gene present in the genome of a selection of *M. mycoides* subsp. *mycoides* SC strains from Table 1, we used the comparative Ct method by performing TaqMan with 10 ng of each genomic DNA and obtaining the corresponding Ct values for both target genes *lppQ* (Ct_lppQ_) and *glpO* (Ct_glpO_). For each strain we then subtracted the Ct values (Ct_glpO_−Ct_lppQ_) to obtain the ΔCt value (normalization of endogenous control). Thereafter, we subtracted the ΔCt of PG1 from that of each strain (ΔCt_strain_−ΔCt_PG1_) to obtain the ΔΔCt value (normalization to PG1). Finally, we used the formula 2^(1+ΔΔCt)^ to estimate the ratio between copies of *lppQ* and copies of *glpO*.
